# COVID-19 in Elderly Patients Receiving Haemodialysis: A Current Review

**DOI:** 10.3390/biomedicines11030926

**Published:** 2023-03-16

**Authors:** Thomas McDonnell, Henry H. L. Wu, Philip A. Kalra, Rajkumar Chinnadurai

**Affiliations:** 1 Department of Renal Medicine, Northern Care Alliance NHS Foundation Trust, Salford M6 8HD, UK; 2 Renal Research Laboratory, Kolling Institute of Medical Research, Royal North Shore Hospital, The University of Sydney, Sydney, NSW 2065, Australia; 3 Faculty of Biology, Medicine & Health, The University of Manchester, Manchester M1 7HR, UK

**Keywords:** Coronavirus 2019, elderly patients, haemodialysis, risk factors, pathophysiology, prevention, management

## Abstract

There is an increased incidence of elderly adults diagnosed with kidney failure as our global aging population continues to expand. Hence, the number of elderly adults indicated for kidney replacement therapy is also increasing simultaneously. Haemodialysis initiation is more commonly observed in comparison to kidney transplantation and peritoneal dialysis for the elderly. The onset of the coronavirus 2019 (COVID-19) pandemic brought new paradigms and insights for the care of this patient population. Elderly patients receiving haemodialysis have been identified as high-risk groups for poor COVID-19 outcomes. Age, immunosenescence, impaired response to COVID-19 vaccination, increased exposure to sources of COVID-19 infection and thrombotic risks during dialysis are key factors which demonstrated significant associations with COVID-19 incidence, severity and mortality for this patient group. Recent findings suggest that preventative measures such as regular screening and, if needed, isolation in COVID-19-positive cases, alongside the fulfillment of COVID-19 vaccination programs is an integral strategy to reduce the number of COVID-19 cases and consequential complications from COVID-19, particularly for high-risk groups such as elderly haemodialysis patients. The COVID-19 pandemic brought about the rapid development and repurposing of a number of medications to treat patients in the viral and inflammatory stages of their disease. However, elderly haemodialysis patients were grossly unrepresented in many of these trials. We review the evidence for contemporary treatments for COVID-19 in this population to provide clinicians with an up-to-date guide. We hope our article increases awareness on the associations and impact of COVID-19 for the elderly haemodialysis population, and encourage research efforts to address knowledge gaps in this topical area.

## 1. Introduction

Emergence of the novel severe acute respiratory syndrome coronavirus 2 (SARS-CoV-2) in December 2019, responsible for the coronavirus 2019 (COVID-19) pandemic outbreak, has since had a profound impact on health systems globally [1]. While it was initially recognized as the cause of severe pneumonias, COVID-19 has since been found to have a range of extrapulmonary manifestations, including direct effects on the kidney [2,3,4,5,6,7]. The pandemic has also disproportionately affected vulnerable populations, such as elderly people and those affected by chronic medical conditions, in which chronic kidney disease (CKD) has been identified as a significant risk factor for morbidity and mortality following COVID-19 infection [8]. In particular, patients receiving haemodialysis face unique challenges, as they require frequent hospital visits and are at risk of COVID-19 exposure from other patients and clinical staff. As a consequence of frailty, multimorbidities and challenges relating to performing dialysis therapy at home, elderly patients requiring kidney replacement therapy are much more likely to receive haemodialysis compared to peritoneal dialysis or kidney transplantation [9]. This review discusses the risk factors and pathophysiological impact of COVID-19 infection in elderly people (i.e., people with age >65 years old) receiving haemodialysis, as well as the practical challenges of managing this patient population when they have a COVID-19 infection. We will explore the current preventative and pharmacological treatment options available to clinicians caring for this vulnerable patient group.

## 2. Risk Factors for Poor Clinical Outcomes in Elderly Haemodialysis Populations with COVID-19 Infection

**Figure 1:**
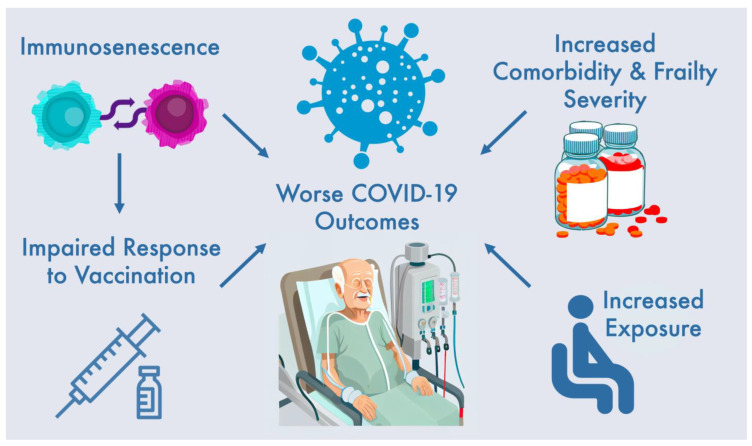
Risk factors for worsened clinical outcomes in elderly haemodialysis patients with COVID-19.

Advancing age is associated with an increased likelihood of contracting COVID-19 infection, with adults above 80 years of age particularly being at greatest risk [8]. In addition, age is a major risk factor for progression towards having acute respiratory distress syndrome (ARDS) [10]. The hazard ratio (HR) for mortality also increases linearly with increasing age [11]. A significantly increased mortality risk is noticeable amongst those with pre-existing kidney disease, particularly those with dialysis-dependent kidney failure. The prevalence of COVID-19 infection is much higher in the CKD population. Outside of old age, CKD patients with an estimated glomerular filtration rate (eGFR) < 30 mL/min/1.73 m^2^ is considered one of the strongest predictors for poor clinical outcomes [11,12]. Mortality rates are exacerbated in the dialysis population over 20 times compared to that expected of propensity-matched historic controls [13]. The risk of death following COVID-19 infection is further increased for an elderly population receiving dialysis [13,14]. There are various reasons which may explain these observed associations (Figure 1).

### 2.1. Increased Comorbidity Status and Frailty Severity

In a vast cohort study undertaken by National Health Service England, nearly 1000 COVID-19-related deaths were reviewed. Age by far was the greatest predictor of mortality, and most comorbidities were associated with increased mortality risk, particularly cardiovascular disease and diabetes. These are both common comorbidities amongst the dialysis population [11]. In another study involving nearly 500,000 dialysis patients in the United States, there were 60,090 patients identified with COVID-19 infection in which the greatest predictor of contracting COVID-19 and increased mortality was nursing home stay, regardless of the length of time the individual had been staying there. Additionally, the number of comorbidities and more importantly, the degree of burden and complications as a result of co-morbid status were all significant risk factors for COVID-19-related mortality [14]. These associations were found in observational studies involving long-term dialysis populations, where increased comorbid status and age correlated with worsened clinical outcomes and mortality rates [12,15]. It is difficult to pinpoint which underlying diseases in haemodialysis patients are more influential in leading towards poor clinical outcomes, due to the multidimensional factors which may contribute towards poor outcomes for this complex group of patients.

Frailty is a significant health burden for elderly patients with advanced CKD and those with kidney failure requiring dialysis treatment [16,17]. Frailty status was included as an important factor in guiding clinical decisions for patient management during early stages of the COVID-19 pandemic [18]. An age-related clinical syndrome characterized by a decline in physiologic reserve and decreased ability to respond to stressor events, frailty is associated with an increased risk of adverse outcomes including falls, hospitalization, poorer health-related quality of life and ultimately, earlier than expected death [19,20,21,22]. Frailty severity is influenced by numerous factors—poor nutrition. sarcopenia, infection and inflammation, cognitive impairment, reduced physical exercise threshold, vitamin D deficiency, metabolic acidosis and cellular senescence are all potential factors which accelerate the decline from fitness to frailty [23,24,25,26,27,28]. Whilst the data in relation to the impact of frailty status and frailty severity on outcomes of elderly haemodialysis patients are only emerging, the presence of COVID-19 infection likely adds further insult to the pathophysiological processes inherent to kidney disease and disrupts homeostatic responses during haemodialysis. These stressors would be more difficult to manage in complex and frail elderly individuals [29,30,31,32]. The management of frail patients receiving haemodialysis is challenging and unfortunately there is no ‘one size fits all’ approach. However, the timely identification of those with poor physical performance levels followed by arrangement of individualized and goal-based exercise programs showed promise [33]. Furthermore, non-pharmacological treatment to aid mood and manage depression has shown positive effects as well [34]. Though malnutrition is often associated with frailty and regular dietary assessments are recommended, there remains a paucity of studies examining the benefits of nutritional supplementation on frailty outcomes in the haemodialysis population at present [34]. Understanding the potential mechanisms of how acute COVID-19 infection affects the elderly dialysis-dependent population may bring further light into the impact of comorbidity and frailty status in this process. The natural history of COVID-19 infection is one manifesting with an initial viral stage, characterized by mild constitutional symptoms, lymphopenia and fever. This may progress to a more malign host inflammatory phase (cytokine storm) associated with ARDS, shock and high C-reactive protein (CRP) activity and the activity of other pro-inflammatory cytokines [35]. This phenomenon is more commonly observed in older age groups, and rarely observed in children and adolescents [36]. Current evidence suggests that elderly patients with COVID-19 infection are more likely to have a higher inflammatory state, with higher CRP levels, lymphopenia, neutrophilia and increased findings of multi lobe lung lesions on Computed Tomography scanning compared to their younger counterparts [37]. It is established that elderly patients and those with kidney failure have higher numbers of CD28 and CD4 null cells and advanced differentiated cells, which may result in the increased likelihood of cytokine storm and acute lung injury [38]. This may also be, in part, secondary to impaired airway clearance and reduced lung reserves in the elderly population affected by comorbidities and frailty.

### 2.2. Immunosenescence

Immunosenescence refers to the changes to the immune system that occur with aging, leading to an increased prevalence of infections, malignancy and autoimmune diseases [39]. Both T and B cells are involved in the process of immunosenescence. The decline in the number and functionality of naive T and B cells leads to an imbalance in the homeostatic mechanisms of the immune system and a decline in the ability to mount a primary immune response to new antigens. This increases the risk of infection and reduces the effectiveness of vaccines in elderly adults. There is a reduction in the number of both pre- and pro-B cells with aging, as well as impairment in the B-cell maturation process and number of immunoglobulins producing B cells [39]. Although there is a significant decrease in the production of B cells from the bone marrow, the number of peripheral B cells will remain constant, reflecting increased B-cell permanence [40]. The thymus is the primary site of T-cell development, and epithelial cells in the thymus are responsible for T-cell development. With advancing age, involution of the thymus is observed where there is a reduction in cellularity and in thymic epithelial space with a consequential decrease in T-cell output [39]. Not only will there be a decline in the number of T lymphocytes (CD4+ T cells and CD8+ T cells) in the circulation, but there will also be a decline in their ability to respond towards incoming pathogens [41]. T-cell clonal exhaustion is also observed, in which there is an impaired T-cell response to new antigens and declining ability to divide and multiply in response to new pathogens [42].

Immunosenescence is likely prevalent within the elderly haemodialysis population [43]. The percentage of newly formed ‘naïve T cells’ is reduced in the circulation with advancing age. This occurs due to a combination of reasons, predominantly explained by the reduction in thymic mass and consequently a reduced output of naïve T cells. Furthermore, the number of differentiated memory T cells increases as we age, and therefore decreases the proportion of naïve T cells in the body. Increased differentiation will also reduce T-cell telomere length [44]. When compared to healthy controls, the T-cell status of patients receiving dialysis is comparable to those up to 20 years more senior in age than themselves [45]. Acceleration in immune aging in the dialysis population is most probably caused by the manifestation of a pro-inflammatory state associated with advanced kidney disease. Accumulation of uremic toxins in kidney failure alongside increased oxidative stress leads to a build-up of pro-inflammatory cytokines such as CD4+ and CD28 as well as CD14+ and CD16++ monocyte populations [43]. The prevalence of senescent and exhausted lymphocyte phenotypes is also markedly increased. This form of accelerated immune aging in pro-inflammatory states, commonly referred to as ‘inflammaging’, explains why the elderly haemodialysis population is more susceptible to risk of infections including COVID-19. A depleted pool of naïve T cells may lead to a slower response to viral inoculation, considering lower numbers of naïve T cells are associated with worsened clinical outcomes and more severe COVID-19 infection [46,47]. Additionally, dialysis patents have a delayed viral clearance of SARS-CoV-2 following the resolution of acute symptoms, with time to a negative polymerase chain reaction (PCR) test being on average 7 days longer for a CKD compared to non-CKD populations [48]. For elderly haemodialysis patients with cytomegalovirus, this has further implications in the context of COVID-19 infection. Given the proportion of CD4 positive and CD28 negative T cells usually make up over 50% of the T-cell population in these groups, the excess pro-inflammatory cytokine activity here can result in greater likelihood of endothelial dysfunction and cytokine storm acutely progressing to systemic sepsis and organ failure if timely intervention is not provided [49].

### 2.3. Reduced Response to COVID-19 Vaccination

Not only are elderly haemodialysis patients at increased risk of contracting and having worse outcomes from COVID-19 infection, but their response to COVID-19 vaccination is also inferior compared with healthy controls [50]. This is an important point to consider, as there has been convincing evidence suggesting protective effects of COVID-19 vaccination with increased antibody levels post-vaccination. Following the first and second COVID-19 vaccinations, healthy controls are expected to mount a threshold serological response 95% and 100% of the time. For dialysis patients, this is significantly reduced, at 45% and 89% [50]. Though for some dialysis patients, the threshold serological response could be reached, the overall antibody titre is still evidently lower in dialysis patients [51]. Considering these findings, elderly haemodialysis patients are at greater risk of poor serological response [52]. A lower antibody titre incurs lower protection during acute COVID-19 infection, particularly from the Omicron variant which is currently the most prevalent COVID-19 variant worldwide [53].

### 2.4. Increased Exposure to COVID-19 Infection

SARS-CoV-2 is a respiratory pathogen, spread via aerosolized droplets. It is highly contagious with an R0 of 2.2 with the degree of risk for infection related to exposure and proximity to an infected individual [15]. Many countries advocated quarantine and isolation measures as primary ways of reducing patient exposure to COVID-19 [54]. With the majority of dialysis-dependent kidney failure patients receiving in-centre haemodialysis, these patients present as a unique group in regard to their increased risk of COVID-19 exposure [55,56,57]. When the COVID-19 pandemic first hit, dialysis centres worldwide had to adapt quickly to protect this vulnerable cohort of patients and employ strategies to reduce their exposure to COVID-19 [58]. The increased risk of contracting COVID-19 and COVID-19 seropositivity amongst patients receiving in-centre haemodialysis is evident, with rates of infection double to those receiving home haemodialysis [59]. A study from the United Kingdom which screened COVID-19 seropositivity status amongst dialysis patients found 36.2% having positive serology (indicating prior infection) and 22.2% having a positive PCR test (though it should be noted that only 21% of the cohort received a PCR test) [60]. The mode of COVID-19 transmission in in-centre haemodialysis patients is predominantly horizontal (i.e., within dialysis shifts where they have interaction with healthcare workers and other dialysis patients) as opposed to vertical (i.e., from the preceding or following shift) [15]. The potential of COVID-19 transmission during shared transport to and from dialysis needs to be considered as another vector of transmission [61]. Though strategies of cohorting and isolating COVID-19 dialysis patients have been implemented during in-centre dialysis, the fact that elderly haemodialysis patients with COVID-19 infection remains seropositive for longer periods hinders the effects from these cohorting and isolation strategies. With over 60% of elderly haemodialysis patients remaining PCR positive for at least 20 days following COVID-19 infection, many of these patients remain infectious for longer periods and are at increased risk of infecting other in-centre haemodialysis patients [62]. Whilst elderly haemodialysis patients remain seropositive for longer, this does not appear to confer adequate protection from re-infection. In the general population with known prior COVID-19 infection, there is a 90% reduction of having a second COVID-19 infection following the first episode, in comparison with a reduction of just 45% for the dialysis population [52].

### 2.5. Risks of Thrombotic Complications

COVID-19 is known to induce a state of hypercoagulability and endothelial dysfunction that correlates with the degree of inflammation [63]. This mechanism does pose an additional health burden for the haemodialysis population who are already at greater risk of thrombosis through exposure to the extracorporeal dialysis circuit. Data on whether there is a significantly increased risk of thrombosis in the elderly population compared to younger persons receiving haemodialysis remain unestablished, but numerous studies have documented catheter, circuit, and arterial venous fistula thrombosis in patients with COVID-19 infection [64,65,66,67]. This can lead to delays in dialysis treatment, excess blood loss and other complications which may impact upon the elderly haemodialysis population more significantly, particularly those living with multimorbidities and more severe levels of frailty [65,66,67]. At the moment, there are no published studies which have evaluated the modification of anticoagulation strategies during intermittent haemodialysis for COVID-19-positive patients, though increasing anticoagulation should be considered during active COVID-19 infection to reduce the risk of thrombosis.

## 3. Prevention Strategies for COVID-19 in Elderly Haemodialysis Patients

There were increased efforts to implement COVID-19 preventative measures specific to elderly haemodialysis patients, due to the increase risk of these patients contracting COVID-19 from their frequent contact and interactions with other dialysis patients and healthcare staff if receiving in-centre dialysis and a high-case fatality from COVID-19 compared to the general population.

### 3.1. Regular Screening

Strategies to prevent patient-to-patient transmission of COVID-19 may include the implementation of regular screening programs. Two types of screening programs can be employed: symptom-based screening followed by specific testing or a universal screening program. The screening process can vary based on the type of test used, such as nasal polymerase chain reaction (PCR), antigen testing or serum antibody testing, and screening programs may also differ based on the frequency of screening.

The International Society of Nephrology COVID-19 guidelines has recommended symptom-based testing as a method to identify and reduce the transmission of COVID-19 in patients with CKD, in particular those receiving dialysis [68]. A study conducted in the United Kingdom during the early phase of the pandemic (from 2 March 2020 to 13 April 2020) reported outcomes from employing a symptom-based testing program. PCR testing was performed if patients reported high-risk symptoms or the presence of fever. Of all the tests performed, 65.6% were positive for SARS-CoV-2. These patients were then separated from asymptomatic patients in an isolation unit. This high proportion of positive tests raises the question of whether symptom-based testing is too insensitive to pick up all COVID-19 infections, particularly since asymptomatic COVID-19 infections have been reported in between 10% and 50% of the dialysis community [60,69,70,71,72].

In another study that used the widely available rapid antigen tests (RAT), 277 haemodialysis patients were screened from 15 February 2021 to 15 November 2021. Thirty-eight tests were returned positive, but only five (14%) cases were subsequently confirmed as COVID-19 positive by PCR testing. Over this period, 6.4% of the haemodialysis population had contracted COVID-19, with routine RAT picking up 27.7% of the cases [73]. A similar study conducted from December 2021 to March 2022 used RAT to screen 220 haemodialysis patients, and 8.5% of asymptomatic patients returned a positive test over this time period (93% were subsequently confirmed COVID-19 positive by PCR testing). Symptomatic patients were screened with a PCR test, and of those who tested positive on PCR, RAT picked up 54% of cases as being COVID-19 positive [74].

Although RAT is able to identify asymptomatic COVID-19-positive cases and is cheaper to perform compared with PCR testing, it may not be sensitive enough to pick up an adequate number of cases. Data on PCR screening programs in haemodialysis populations are less well documented, but in one study, 200 patients were screened using PCR testing over a five-day period in early 2020 in a dialysis unit in France. Of these, 19% of patients had a positive result returned from their PCR test, and of those with a positive test result, 10% were asymptomatic [70]. If readily available, a universal PCR test screening program is more likely to pick up a greater number of COVID-19 cases than RAT, though the imperfect nature of PCR testing to identify COVID-19 cases has also been considered. One study sampled serum samples of 356 patients receiving dialysis, in which 129 patients were SARS-CoV-2 antibody positive. In this patient cohort, 40% of patients were asymptomatic from COVID-19 symptoms and of the 42 patients who had a negative PCR test result, eight were SARS-CoV-2 antibody positive [72].

It should be acknowledged that the clinical phenotype of COVID-19 has changed over time through the natural history and evolution of the virus and the increased delivery of vaccination. Esposito and colleagues highlighted that a cohort of COVID-19 haemodialysis patients in September 2021–February 2022, when compared to COVID-19 haemodialysis patients in March-December 2020 experienced less severe illness, though there were greater frequencies of asymptomatic disease [75]. This has likely impacted on the effectiveness of symptom-based screening programs. Ultimately, the cost-effectiveness of a screening system relies not only on the sensitivity of the test used, but also on the prevalence of the disease in focus. During the peak points of COVID-19 prevalence, a symptom-based screening program is unlikely to be adequate enough to capture all cases due to the high prevalence of asymptomatic COVID-19 infection. This may be adequate in times of low COVID-19 prevalence, though a dynamic and responsive screening system adapted to understanding the local prevalence of COVID-19 infection would be ideal. This philosophy of implementing COVID-19 screening strategies has been endorsed by many health systems, in which weekly PCR testing was recommended in areas where there are available resources during times of high community prevalence of COVID-19 [76].

### 3.2. Cohorting and Isolation Strategies

Once COVID-19-positive individuals receiving haemodialysis have been identified, it is important to cohort infected patients together to prevent a further spread of COVID-19 around the dialysis unit. The timeframe which presents the greatest risk of COVID-19 infection spread is within a dialysis session in which there are COVID-19-positive patients, in which horizontal transmission is commonly observed particularly in small in-centre dialysis venues where there are the greatest rates of infectious transmission [15]. There are variable reports in regard to the impact of shared transport to and from in-centre dialysis as a vector of COVID-19 infection spread. Some studies noted a significant association whilst other studies have not, though it has been recommended that patients should use private transport to and from in-centre dialysis if possible [15,77]. It is acknowledged that this may be challenging for many elderly haemodialysis patients who are unable to drive to and from in-centre dialysis due to their functional limitations, and many may not have family support to transport them to and from care facilities for dialysis [78].

The time recommended for elderly haemodialysis patients to remain isolated if COVID-19-positive test results were found is also longer than the general population. This is based on findings that the average time to a first negative test for kidney failure patients is 7 days longer than someone without kidney failure [48]. Over 65% of dialysis patients have a positive COVID-19 PCR test at 20 days [62]. Given increasing age has been noted to be an independent factor for lengthier viral clearance, it is suggested that the time spent on quarantine should be longer for these patient groups, or until a negative PCR test result is established [79]. It is difficult to prove whether positive COVID-19 PCR test results represent an active, transmissible infectious process or is simply reflecting the legacy of prior disease. Karoui and colleagues recommend the discontinuation of patient isolation once their COVID-19 vital load is lower than <1,000,000 copies/mL [52].

### 3.3. COVID-19 Vaccination

While most haemodialysis patients experience a durable immune response following a full COVID-19 vaccination schedule that is comparable to the general population, there is evidence to suggest that those with higher initial antibody titres exhibit a more prolonged response [80,81,82,83]. When reviewed after a second vaccination dose, reduced responses have been observed in haemodialysis patients as indicated by lower peak antibody levels compared to the general population [46,82]. A lower level of immunity is greatest amongst elderly individuals receiving haemodialysis [46,82]. However, a third COVID-19 vaccination dose has been demonstrated to have elicited increased and adequate peak antibody levels for this patient group [84]. These findings underscore the importance of ongoing surveillance for immune responses to COVID-19 vaccination in elderly haemodialysis patients, and highlights the clinical advantages of a third COVID-19 vaccination dose for them. Ongoing work in this area may inform future vaccination strategies for this vulnerable population.

In the current landscape of vaccine development for the prevention of COVID-19, two principal categories of vaccines have emerged: mRNA vaccines and adenovirus vector vaccines. The former includes the BNT162b2 (Pfizer-BioNTech) and mRNA-1273 vaccines (Moderna), while the latter encompasses the AZD1222 (AstraZeneca) and Ad26.COV2.S vaccines (Johnson & Johnson). The mRNA vaccines function by delivering genetic instructions for producing the viral spike protein to the host cell through the use of lipid nanoparticles, while the adenovirus vector vaccines rely on the use of modified adenoviruses to deliver genetic material for spike protein production to the host cell. Though both types of vaccines have demonstrated efficacy and safety in clinical trials involving the general population, and their widespread use has played a pivotal role in mitigating the ongoing COVID-19 pandemic, emerging data suggest that antibody responses to COVID-19 vaccination may differ amongst the various vaccination brands in elderly haemodialysis patients.

The adenovirus vector vaccine AZD1222 (AstraZeneca) has demonstrated reduced immunogenicity in patients receiving haemodialysis, in comparison to the mRNA BNT162b2 (Pfizer-BioNTech) vaccine [85]. Whilst three doses of the BNT162b2 vaccine have demonstrated adequate antibody response against the Omicron variant (the most prevalent strain of COVID-19 at present), the threshold is met in only 50% of cases when this vaccine is given to haemodialysis patients as a booster on top of two prior administered AZD1222 (AstraZeneca) vaccines [86]. Additionally, antibody responses are lower in haemodialysis patients given the adenovirus vector Ad26.COV2.S (Johnson & Johnson) vaccine compared to either mRNA vaccines [47]. Regardless, it would seem appropriate to vaccinate elderly haemodialysis patients with an mRNA COVID-19 vaccine. If either mRNA vaccine is available, then the mRNA-1273 (Moderna) appears to be the superior option, though a higher dose of the BNT162b2 (Pfizer-BioNTech) vaccine may also be suitable for dialysis patients in its absence.

In regard to the frequency of COVID-19 vaccination for elderly haemodialysis patients, previous studies have demonstrated that antibody levels in the general population following COVID-19 vaccination begin to decline approximately six months post-inoculation, with subsequent increased risk of infection but providing continued protection against risk of complications leading to hospitalization and mortality [87,88]. There is evidence of an earlier and more significant decrease in immunogenicity amongst dialysis patients, despite previous investigations not including matched control comparisons [89]. De Vriese and colleagues conducted a comparative study between responses to the BNT162b2 (Pfizer-BioNTech) and mRNA-1273 (Moderna) vaccines in dialysis-dependent and healthy control groups. Their study revealed suboptimal humoral and cellular immunities in dialysis patients at 24 weeks. While initial responses were stronger in the mRNA-1273 vaccine compared to the BNT162b2 vaccine, the cellular response was still attenuated at 24 weeks in haemodialysis patients. Notably, those with prior exposure to COVID-19 exhibited the greatest antibody response, with the vaccine brand received concluded to be of secondary importance [90]. This waning of antibody titres at an earlier point in the dialysis population raises awareness to consider more frequent booster vaccination programs, to prevent increased risks of COVID-19 infection in an at-risk patient population such as elderly persons receiving haemodialysis.

## 4. Treatment Strategies for Elderly Haemodialysis Patients Infected with COVID-19

Numerous medications have been proposed or developed during the pandemic in an effort to find treatment options for COVID-19, and to explore how patient outcomes could be improved. In a broad sense, therapeutics could be classified into two categories: antiviral and anti-inflammatory treatments. Antiviral treatments are aimed towards patients in the pre-hospital setting, during the early viral replication phases of their disease course. In contrast, anti-inflammatory treatments are intended for patients acutely unwell in the hospital setting, typically those requiring oxygen therapy, and those who exhibit signs of pneumonitis, with the goal of attenuating the cytokine/hyperinflammatory response. Due to the rapid emergence and evolution of COVID-19 pandemic data, discussion of all antiviral treatments that have been developed during the pandemic is beyond the scope of our review, as many antiviral treatments were eventually found to be ineffective and the Omicron variant with its numerous mutations in the spike protein has rendered many previous treatments obsolete. We will focus on treatment options that have received approval from the Centers for Disease Control and Prevention (CDC), the National Institutes of Health (NIH) and the National Institute for Health and Care Excellence (NICE) and discuss key trial findings, as well as evidence in relation to their use, efficacy and adverse effects within the context of elderly patients, patients with kidney disease and those receiving haemodialysis (Table 1).

### 4.1. Nirmatrelvir and Ritonavir (Paxlovid)

Nirmatrelvir and ritonavir (Paxlovid) is a boosted 3cl protease inhibitor. It has been trialed in the EPIC-HR study which was a randomized trial which administered 300 mg of oral nirmatrelvir and 100 mg of oral ritonavir every 12 h for 5 days, or a placebo in non-vaccinated COVID-19-positive patients pre-hospital admission. The primary outcome of the study was risk of hospital admission or death from any cause [91]. The study included a provisory that patients had to have at least one established risk factor for developing severe disease following COVID-19 infection, in which the list of risk factors included age > 60 years old and CKD. However, the EPIC-HR study did not describe the proportion of dialysis patients involved, nor provided data on eGFR from the included patients. Though the median age of study participants was 45 years old, when subgroup analysis was completed for those > 65 years old, effect size did appear to have increased in elderly patients. Haemodialysis is unlikely to significantly clear nirmatrelvir from the circulation. To achieve effective blood concentrations for enzyme inhibition, a dose of 300 mg nirmatrelvir (with 100 mg ritonavir) on day 1, followed by 150 mg nirmatrelvir (with 100 mg ritonavir) administered daily after haemodialysis during dialysis days has been proposed [92,93]. However, as nirmatrelvir is a potent inhibitor of CYP3A4, caution should be exercised when administering it to patients receiving medications and other treatments which are metabolized by this enzyme. It is of note that kidney transplant recipients taking tacrolimus may have nirmatrelvir levels 10-fold higher than normal levels, and careful evaluation of the risks and benefits in this context is needed [92]. The PANORAMIC trial is an ongoing study which aims to assess the benefits of nirmatrelvir and ritonavir for those testing positive for COVID-19, and is open towards recruiting patients with CKD and kidney failure including those who are dialysis dependent [94].

### 4.2. Remdesivir

Remdesivir functions via its incorporation into viral RNA and termination of RNA transcription. This drug was trialed in the PINETREE study, a randomized controlled trial in which intravenous remdesivir (200 mg on day 1 and 100 mg daily on days 2 and 3) was compared with a placebo in non-vaccinated patients in pre-hospital settings [95]. The primary outcome was determining whether remdesivir reduced risk of hospital admission or death from any cause. Similar to the previous trial for nirmatrelvir and ritonavir, the included patients had to have at least one risk factor for developing severe disease following COVID-19 infection, which includes age > 60 and CKD. It was documented in the PINETREE study that only 2.5% of study participants had CKD whilst there were no dialysis patients included. The mean age was 50 years old. The effect size of remdesivir when compared to nirmatrelvir and ritonavir appears to be less, whilst another disadvantage of the drug is that it could only be administered as an intravenous preparation.

In another retrospective cohort study conducted between January and March 2022, 118 haemodialysis inpatients with positive COVID-19 tests were evaluated [96]. The mean age of the cohort was 68.5 ± 12.8 years. A total of 44 patients (37.3%) were administered with a loading dose of 100 mg and a maintenance dose of 50 mg for the next 2 to 4 days post-dialysis during dialysis days. The authors found that the remdesivir group had a lower risk of composite mortality and aggravation of disease severity, despite a higher level of disease severity at hospitalization compared to the non-remdesivir group. More importantly, there were no statistically significant differences between the two groups in terms of adverse events related to treatment. It should be considered that this study was retrospective in nature and as such, it may be subject to certain limitations, including potential bias in the selection of patients and incomplete data. Ultimately, study findings seem to suggest that remdesivir may be safely administered as an alternative option if nirmatrelvir and ritonavir is not available or when the patient is taking a calcineurin inhibitor.

### 4.3. Molnupinavir

Molnupinavir is a prodrug of N-hydroxycytidine (NHC), a nucleoside analogue. It has been trialed in two randomized clinical trials—the MOVe-OUT trial and another study by Fischer and colleagues compared clinical outcomes in pre-hospital non-vaccinated patients taking 800 mg of molnupiravir for 5 days versus placebo [97,98]. The primary outcome was the risk of hospitalization or death from any cause. There were some differences in the conclusions of these two studies, with the MOVe-OUT trial displaying improvement in the primary outcome with molnupiravir use whilst the study by Fischer and colleagues only found improvements in reducing viral load. Amongst the MOVe-OUT trial study participants who received molnupiravir, only 5.3% were diagnosed with CKD in which patients on haemodialysis were excluded from study participation. The median age of study participants in the MOVe-OUT trial was 42 years old. There were no statistically significant differences in study outcomes between molnupiravir and placebo treatment in a sub-group analysis for patients > 60 years old.

There are few studies which investigated the efficacy of molnupiravir in the context of the dialysis population. Poznansk and colleagues presented a retrospective cohort study reporting on the use of molnupiravir in 20 dialysis patients with positive COVID-19 test results [99]. These patients received a regimen of 800 mg of molnupiravir twice daily for 5 days. Their study also included an additional 16 transplant patients, who did not experience any serious side effects or drug interactions with their immunosuppressive therapy. The authors noted that the symptoms of COVID-19 amongst the dialysis patients improved rapidly or resolved within 24–48 h of starting treatment, though there were no matched controls in this study. Further investigation on the use of molnupiravir in the dialysis setting is required, though initial results are promising for its use as a safe home therapy for haemodialysis patients with positive COVID-19 status including those receiving simultaneous immunosuppressive treatment.

### 4.4. Dexamethasone

The RECOVERY trial aimed to evaluate the effectiveness of administering 6mg of dexamethasone for 10 days to adult patients diagnosed with COVID-19 infection [100]. Among adult patients requiring supplemental oxygen, corticosteroid treatment was shown to decrease all-cause mortality, increase hospital discharge rates, and potentially reduce the need for invasive mechanical ventilation and mortality within 28 days of treatment initiation compared to usual care or a placebo. The mean age of the study population was 66.9 ± 15.4 years, with 23% of patients being over the age of 80. Patients undergoing haemodialysis were excluded from the RECOVERY trial, although 8% had an eGFR <30 mL/min/1.73 m^2^. Another retrospective analysis of haemodialysis patients with COVID-19 infection receiving dexamethasone did not demonstrate a clear clinical benefit [101]. However, this retrospective study was limited by its methodology, and it remains in current recommendations that haemodialysis patients with COVID-19 infection requiring oxygen support should receive steroid treatment.

### 4.5. IL-6 Inhibitors—Tocilizumab and Sarilumab

The interleukin-6 (IL-6) inhibitors, tocilizumab and sarilumab, have demonstrated reductions in all-cause mortality amongst hospitalized adults with COVID-19 when used with the standard care that was described in the RECOVERY and REMAP-CAP trials [102,103]. Tocilizumab and sarilumab is recommended for patients hospitalized with COVID-19 who require oxygen and have evidence of severe inflammation, as defined by CRP levels >75 or rapidly increasing oxygen requirements. However, the use of these IL-6 inhibitors comes with a significant risk of bacterial infection, and patients with suspected bacterial infection should not receive IL-6 inhibitors. In REMAP-CAP, the mean age of the study participants was 61.5 ± 12.5 years, and in RECOVERY, this was 63.3 ± 13.7 years, with 11% of patients aged >80 years. In REMAP-CAP, 9.6% of patients were labeled as having CKD, but no information on eGFR or dialysis dependency was provided. Current data on the use of IL-6 inhibitors for treating dialysis patients with positive COVID-19 status are limited, although a study from Japan assessing tocilizumab in patients with kidney failure and rheumatoid arthritis has demonstrated an acceptable safety profile [104]. Further work is needed to validate the consideration of IL-6 inhibitor use for this patient population, particularly elderly patients receiving dialysis.

### 4.6. Baricitinib

Baricitinib, a Janus kinase (JAK) inhibitor, was shown to be beneficial for hospitalized patients diagnosed with COVID-19 infection in the RECOVERY trial [105]. Importantly, clinical benefit was observed if baricitinib was administered in addition to steroids and IL-6 inhibitors, and the majority of patients (95% of patients) were already receiving steroids whilst 23% received tocilizumab. The mean age of the study cohort was 61.5 ± 12.5 years, with 8% being age >80 years. Only 2% had an eGFR < 30 mL/min/1.73 m^2^ and unfortunately, the use of baricitinib is currently not recommended for patients on dialysis. A phase 2 trial is currently underway to investigate its use in patients with diabetic kidney disease [106].

### 4.7. Other Novel Therapies

The PROTECT-V (PROphylaxis for paTiEnts at risk of COVID-19 infecTion) study is a clinical trial aimed at evaluating the effectiveness of intranasal niclosamide prophylaxis in preventing COVID-19 infection [107]. Niclosamide, originally used for the treatment of tapeworm, has been shown to exhibit antiviral activity against SARS-CoV-2 in cell culture studies. The PROTECT-V trial will also evaluate the effectiveness of sotrovimab, a human IgG1κ monoclonal antibody that targets the spike protein of SARS-CoV-2, which is administered as a single infusion. This study is actively recruiting dialysis patients, given their increased risk of having poor clinical outcomes following acute COVID-19 infection is more well established, though they have previously been excluded from large-scale clinical trials.

## 5. Conclusions

**Figure 2:**
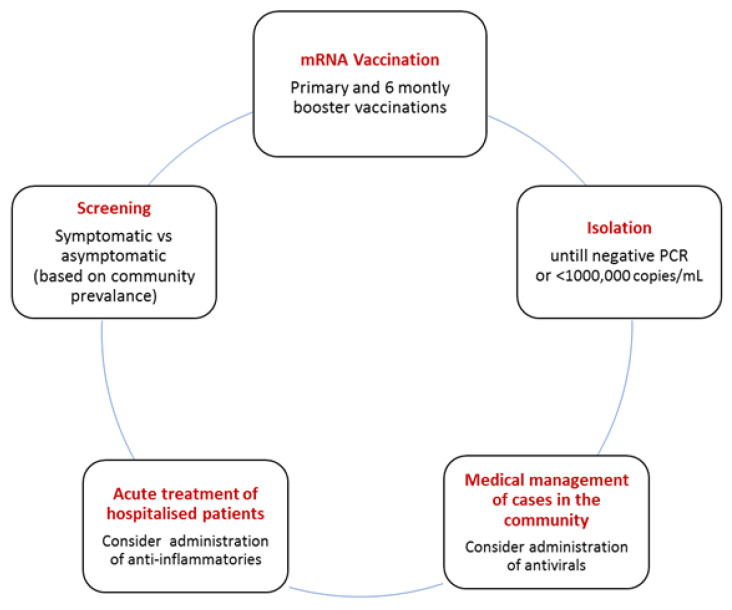
A pragmatic COVID-19 management approach for elderly patients receiving haemodialysis.

Evidence regarding the associations between COVID-19 and clinical outcomes in dialysis patients, particularly the elderly haemodialysis population, has certainly increased since the onset of the pandemic. Our understanding of the various factors which contribute towards increased risks of contracting COVID-19 infection and disease severity for this vulnerable patient population has been broadened with the emergence of basic and clinical trial research within this topical area. Widening the recruitment and sub-study of the elderly haemodialysis population in ongoing COVID-19 clinical trials over time may further inform the long-term implications of COVID-19 for this patient group. It is encouraging that at a local, national and international level, there have been preliminary data to establish directive guidance for the prevention and medical treatment of COVID-19 infection in dialysis patients. Figure 2 outlines components of a pragmatic COVID-19 management approach for elderly patients receiving haemodialysis considering the currently available evidence. Nevertheless, there remains limited data in regard to the efficacy and adverse event profile of measures specifically for the elderly haemodialysis cohort. Going forward, further studies considering the unique challenges faced by these individuals compared to the general population and kidney disease patients who have received or are receiving other forms of kidney replacement therapy are required.

## Figures and Tables

**Table 1:** Summary of potential antiviral and anti-inflammatory treatment options for elderly haemodialysis populations with COVID-19.

Antiviral Treatment (Administered in Pre-Hospital Patients Not on O_2_ Therapy)					
Drug	Mechanism of Action	Outcome	Key Trials	Mean Age (years) of Study Participants in Key Trials	Recently Published/Ongoing Trials Studying for Use in Haemodialysis Populations
Nirmatrelvir and ritonavir (Paxlovid)	Boosted 3cl protease inhibitor	Reduced risk of hospitalisation for COVID-19 or death from any cause	EPIC-HR (2022)	45.0 (18−86)	PANORAMIC (currently recruiting)
Remdesivir	Incorporation into viral RNA and termination of RNA transcription.	COVID-19-related hospitalisation	PINETREE (2022)	50.0 ± 15.0	Jeong-Hoon and colleagues (2022)
Molnupiravir	Prodrug of N-hydroxycytidine (NHC), a nucleoside analogue	Reduced the risk of hospitalisation or death from any cause	MOVe-OUT (2022) Fischer (2021)	42.0 (18−90)	Poznansk and colleagues (2022)
Anti-inflammatory treatment (administered in hospitalized patients)					
Dexamethasone	Glucocorticoid	Reduced all-cause mortality and discharge from hospital.	RECOVERY trial (2021)	66.9 ± 15.4	-
Tocilizumab/sarilumab	IL-6 inhibitors	Reduced all-cause mortality	REMAP-CAP (2021) RECOVERY (2021)	61.5 ± 12.5 63.3 ± 13.7	-
Baricitinib	JAK inhibitor	Reduced mortality and progression to invasive mechanical ventilation	RECOVERY (2022)	58.5 ± 15.4	Drug is not recommended for patients receiving dialysis

## Data Availability

No new data were created for this article.
